# Craving for exercise due to the disruption of daily routine by an earthquake

**DOI:** 10.3389/fspor.2024.1508317

**Published:** 2025-01-14

**Authors:** İsmail İlbak, Ahmet Yasuntimur, Stefan Stojanovic, Serkan Düz, Łukasz Rydzik, Ladislav Cepicka

**Affiliations:** ^1^ Institute of Health Sciences, İnönü University, Malatya, Türkiye; ^2^ Institute of Social Sciences, İnönü University, Malatya, Türkiye; ^3^ Faculty of Sport and Physical Education, University of Niš, Niš, Serbia; ^4^ Faculty of Sport Sciences, İnönü University, Malatya, Türkiye; ^5^ Institute of Sports Sciences, University of Physical Education, Krakow, Poland; ^6^ Department of Physical Education and Sport, University of West Bohemia, Pilsen, Czech Republic

**Keywords:** craving for exercise, interruption of physical exercise, earthquake, craving due to addiction, craving due to anxiety

## Abstract

**Objectives:**

The research aimed to examine exercise cravings that arise from the disruption of daily routine resulting from the earthquake and the consequent mandatory absence from exercising at fitness centers.

**Study design:**

This study utilized the phenomenological design through qualitative research methods.

**Methods:**

The research sample comprises six individuals aged between 22 and 29 years. They were selected using the criterion sampling method. Data was collected through semi-structured interviews, and thematic analysis was used to analyze the data.

**Results:**

Two different types of cravings were identified. The first type is related to exercise addiction, while the second type is related to anxiety-induced cravings.

**Conclusions:**

Exercise craving manifests in two ways. The first type originates from exercise addiction, emerging when an individual with exercise addiction experiences a lack of exercise. With a more clinical dimension, the second type arises from the inability to engage in training for uncontrollable reasons.

## 1 Introduction

Individuals, as a result of their vital life experiences throughout the historical process, exhibit different levels of excitement and interest toward particular objects, facts, materials, and behaviors. This excitement and interest can eventually lead to addiction by imprisoning individuals over time. Addiction is a compelling and recurring need that forces individuals and, if left unfulfilled, can result in negative consequences that are strong and easily preventable (1). When reviewing the literature, it has been reported that behaviors such as internet and social media usage (2), sex (3), eating (4), and exercise (5) can be addictive. However, in behavioral addiction, it is generally known that craving occurs before addictive behavior (6). Therefore, it is believed that craving plays a critical role in the occurrence and maintenance of addictive behavior (2). The pleasure experienced by an individual following a behavior may lead to craving for that behavior, and depending on the frequency of craving and the frequency of its resolution, addictive behavior can develop.

Craving, first defined in 1955 as a difficult-to-understand component with a repetitive effect (7), refers to a strong desire, impulse, and urge (8). Although craving is generally used for substance use, it is not limited to substance use only, and it is known to be frequently used in terms of concept, definition, and usability by researchers from different disciplines (6). Particularly in recent years, it has been observed that the concept of craving has started to play an important role in behavioral cravings (9). Behavioral and substance addictions share phenomenological similarities. Individuals experiencing behavioral addictions often describe a pre-behavioral urge or craving, analogous to individuals with substance use disorders experiencing cravings before substance use. Moreover, these behaviors frequently alleviate anxiety and induce a positive emotional state or a sensation akin to substance intoxication (6, 10). When the literature is examined, studies on cravings for food (4), gambling (10), and travel (9, 11) are found to be available. However, despite exercise addiction being a behavioral addiction, the exact role of the concept of craving is not well understood.

Individuals who regularly engage in sports or exercise may occasionally experience unexpected events, such as a heavy workload, injury, lack of motivation, pandemic-related restrictions, and natural disasters like earthquakes, which can cause them to stay away from exercise temporarily. As a result, individuals may feel a strong desire to exercise. The situation where exercise is intensely desired during this period when exercise cannot be performed is defined as exercise craving.

In this context, life in eleven provinces has stopped because of two major earthquakes with magnitudes of 7.7 and 7.6, which occurred on February 6, 2023, in Türkiye, and many people and structures have been adversely affected. Since life will take a long time to return to normal in these provinces, many individuals have had to live outside of their regular routines. Therefore, this study aims to examine the exercise cravings of individuals who have been forced to stay away from sports facilities and exercise for a certain period of time due to the earthquake disaster.

## 2 Materials and method

### 2.1 Research design

This study utilized the phenomenological design (12, 13) through qualitative research methods. The participant group was selected using convenience sampling (14). Data was collected through semi-structured interviews (15), and thematic analysis was used to analyze the data (16). The findings were presented in a report format. This research was carried out per the Declaration of Helsinki after being approved by the Scientific Research and Publication Ethics Committee of İnönü University (Approval number: 2023/4508).

### 2.2 Study group

The participants of this study were selected using the criterion sampling method. In qualitative research, large sample groups are not needed as data collected through interview and observation techniques may repeat itself after a while. The point at which data starts to repeat itself is called the saturation point, and data collection can be terminated (17, 18). Therefore, it was decided that the research group would consist of 6 individuals as data started to repeat itself after the 6th participant. All participants were male residing in Malatya province and experiencing the earthquake. The participants who agreed to participate were included in the study after reading and signing the informed consent form. In reporting research findings, participants’ names were kept confidential, and alphabetical and numerical symbols such as A1, A2, A3, A4, A5, and A6 were used instead of names. The demographic characteristics of the participants are shown in Table 1.

**Table 1:** Demographic characteristics of participants.

Participant	Age	Marital status	Exercise experience (years)	Exercise frequency (days/week)
A1	24	Single	4	5
A2	22	Single	3	3
A3	27	Single	8	3
A4	25	Single	5	5
A5	29	Single	8	6
A6	26	Single	4	5

The youngest participant was 22 years old, and the oldest was 29. All participants were single. The participant with the most extensive exercise experience had been exercising regularly for eight years. The participant with the highest weekly exercise frequency exercised six days a week.

### 2.3 Inclusion criteria

The inclusion criteria for the study are as follows:

• Engaging in regular exercise for at least six months before earthquakes
• Exercising at least three days per week in a gym before earthquakes
• Not having engaged in exercise since the earthquakes that affected Malatya, Türkiye on February 6, 2023.

### 2.4 Data collection tools

This study used a semi-structured interview form (15) as the data collection tool. The opinions of three field experts and two measurement evaluation experts were obtained to ensure the validity of the interview form. Data was collected on February 26th, 2023, in Malatya, Türkiye. Interpretive approaches, which conceptualize qualitative methods (13), were utilized in collecting data. To ensure a comfortable conversation with the participants, warm-up questions unrelated to the study's main aim (19) were used to start the interview. The main questions related to the research aim were included in the interview form. Moreover, trigger questions based on the naturalistic inquiry approach were asked (20) when necessary to examine the topic in depth (15). All interviews were recorded using a voice recording application on a mobile phone with an Android operating system. The interview recordings were compiled into a single text and prepared for thematic analysis.

### 2.5 Data analysis and reliability

**Figure 1:**
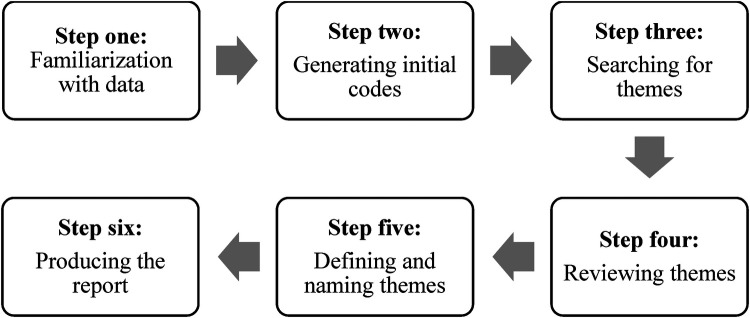
Six-Step thematic analysis technique.

This study used a thematic analysis technique to analyze the data. Thematic analysis is a highly effective method for analyzing, defining, and reporting themes from a dataset (16), so this research preferred the thematic analysis technique. The six-step thematic analysis technique is shown in Figure 1.

Several strategies were employed to enhance the research's credibility:

• The various stages of the research process were explicitly outlined.
• Ambiguous statements were clarified in certain instances, and the consistency of the gathered data was guaranteed by rephrasing identical questions in different manners.
• The data collected from participants was scrutinized, and participants’ statements were cross-verified.

## 3 Results

Two themes emerged from the data obtained from the participants**’** discourses in the study (Table 2).

**Table 2:** Themes developed from the participants’ discourses.

Theme 1: Craving due to exercise addiction
Theme 2: Craving due to anxiety

### 3.1 Theme 1: craving due to exercise addiction

Participants expressed a common view that they were craving to do exercise because they perceived themselves as exercise addicts. This type of craving develops after exercise addiction.

“I have been exercising for years and continue to do so with great dedication. Even if I miss a day of exercise, I feel quite uncomfortable and guilty. I can easily say that I am an exercise addict. Not being able to do exercise is very sad, and this situation never leaves my mind. I cannot tell you how often I want to exercise clearly a day because it is always on my mind” (Participant A1).

“I am definitely an exercise addict. I have been doing exercise without missing a single day for years. I had exercised at around 5 or 6 pm for over a year before earthquakes. I can say that my desire to exercise reaches its peak, especially during those hours. However, if I generalize, I can say that the thought of doing exercise is on my mind for about 70% of the day” (Participant A2).

“I have been exercising since a very young age. However, after graduating from high school, I started doing fitness. Due to the changes in my physical appearance, everyone's perception of me changed positively. This motivated me to engage in fitness even more. Then, I continued doing fitness regularly. It has become something like eating and drinking for me. When I don't exercise, I feel bad. If I skip exercising for a day, I feel as if I haven't done anything at all that day” (Participant A3).

“Doing exercise makes me happy. I feel very good when I do exercise. I think I am definitely an exercise addict. I feel the sadness of not being able to do exercise all day long. I cannot tell you how often it comes to my mind, but I can say that it is on my mind all day long” (Participant A4).

“I do not doubt that I am an exercise addict. I have been enthusiastically exercising at the gym for years and enjoy it very much. Knowing that I cannot exercise keeps this situation in my mind. I sincerely want to state that I desire to exercise frequently during the day” (Participant A5).

“I do not know whether being an exercise addict is a positive thing, but I can happily say that I am an exercise addict. I always need to exercise, and I am not complaining about this situation. I have been exercising at around 8 pm for the last seven months. It bothers me a lot to not be able to do exercise, especially when my workout time approaches. However, during the day, this situation comes to my mind occasionally, and it does bother me” (Participant A6).

### 3.2 Theme 2: craving due to anxiety

All participants expressed a common view that they were craving because they were worried that their physical appearance would be negatively affected by being unable to exercise.

“Not being able to do exercise worries me a lot. Because I have been doing exercise for years and I think I have a very good physical appearance, I do not want to lose this form. Not being able to do exercise worries me a lot. Because I have been doing exercise for years and I think I have a very good physical appearance, I do not want to lose this form. That is why I feared losing my form after the earthquake. For the first time, I could not exercise for a long time and felt like exercising a lot” (Participant A1).

“I have been exercising for a long time and have attained an excellent physique. I am concerned that my muscles might shrink, intensifying a persistent craving fueled by the anxiety that without regular workouts, I could lose not only physical strength but also the mental resilience that exercise has become for me. This leaves an unmet need for the therapeutic release it provides during times of stress” (Participant A2).

“Not being able to do exercise is very painful. I gain weight very quickly when I do not exercise. I am very afraid of gaining weight because I cannot exercise right now. Doing exercise is always on my mind because I constantly think about it, knowing that I cannot do it, and this situation is disturbing” (Participant A3).

“I go to do exercise with my friends, and we constantly compete to see who can do Bench Press with the heaviest weight. I am worried I will lose my previous strength” (Participant A4).

“I am very restless because I cannot do exercise. I hope I do not lose the physique and strength I have. I want life to return to normal immediately because I have trained so much to get this physique, and the thought of losing this physique keeps running through my head” (Participant A5).

“Of course, not being able to do exercise is very sad. I have been doing exercise for many years, and I have gained a very good muscle mass. I have a great concern about whether I will lose my muscle mass. The fear of losing muscle mass makes me feel as if I am deprived of a basic need, intensifying my craving for exercising” (Participant A6).

## 4 Discussion

This study aimed to examine the phenomenon of exercise craving among individuals who could not exercise due to disrupting their normal routines following the two major earthquakes with magnitudes of 7.7 and 7.6, which occurred in Türkiye on February 06, 2023. The feelings of individuals unable to exercise were deeply analyzed based on their statements. Two types of cravings were found in the research. The first one arises from the exercise addiction of individuals who define themselves as exercise addicts. Exercise addiction is defined as the state in which exercise is out of the individual's control, and the duration, frequency, and intensity of exercise are continuously increased to achieve the desired effect of exercise (21, 22). In this type of craving, the individual who cannot exercise after the earthquake suffers from exercise deprivation. It can be stated that craving, which occurs in the form of intense desire and strong impulses in exercise addicts, is caused by the deprivation of a habit.

As in other behavioral addictions (2, 4, 10) individuals with exercise addiction also exhibit typical exercise cravings (5). In other behavioral addictions, when the addictive behavior does not recur due to uncontrollable reasons, an urge or motivation to engage in the behavior emerges in the individual (21). Therefore, the current research findings align with and parallel previous research results, supporting each other.

According to the research findings, the second type of craving occurs due to anxiety. In this study, all participants who identified themselves as exercise addicts expressed concern about the negative effects of exercise deprivation on their physical appearance and athletic performance levels.

Craving is a strong desire that can cause a person to engage in negative behaviors or become obsessed with satisfying them (22). This is because the craving becomes a necessity for the craving person, just like eating (23). Therefore, it is thought that cravings may be the underlying cause of the anxiety caused by not being able to exercise.

Previous research has revealed different results. Kavanagh et al. noted that craving, which appears to be related to factors such as environment, expectations, mood, self-efficacy, and intentions, can cause distress and discomfort, especially during deprivation attempts (24). Individuals with exercise addiction (25–27) have expressed overcoming the desire to relapse into behavioral addiction due to withdrawal after a certain period, similar to individuals with other behavioral addictions (3, 9, 11). Research on exercise addiction suggests that when exercise is not performed, withdrawal symptoms manifest as psychological indicators such as stress, depression, and anxiety (28–31). Therefore, in behavioral addictions, the emergence of anxiety as a sign of withdrawal is commonly accepted.

However, our research findings provide a more nuanced perspective, indicating that exercise cravings based on anxiety in individuals with exercise addiction are not solely derived from exercise dependency. This suggests that additional factors, such as physiological changes, may play a significant role in shaping these cravings. For instance, elevated cortisol levels, a stress hormone, during exercise deprivation can heighten anxiety and depressive symptoms (32). Similarly, reductions in reproductive hormones like testosterone have been linked to addictive behaviors (33). Neurotransmitter disruptions, such as changes in dopamine and endorphin systems, also contribute to addiction-like behaviors, reinforcing the parallels between exercise and substance addictions (34). Dysregulation of the hypothalamic-pituitary-adrenal (HPA) axis further complicates stress responses and addiction mechanisms (35). These findings underscore the multifaceted physiological and psychological dynamics underlying exercise addiction and its cravings.

Based on the interviews with the participants, it can be argued that psychological factors are closely intertwined with physiological mechanisms. Specifically, this type of craving may be caused by muscle dysmorphia (MD) or may lead to MD in advanced stages. When specifically examining muscle dysmorphia, the individual's attention is directed toward the insufficient size and muscularity of their body (36, 37). Participants expressed concerns about a potential decrease in their current physical appearances, specifically in muscle mass, as they anticipate being unable to engage in training post-earthquake. Consequently, they indicate a strong motivation to engage in high levels of exercise.

This study has several limitations, which are outlined below. The small sample size, limited to only six participants, restricts the generalizability of the findings. Additionally, the participants were all within the 22–29 age range and were single, which limits the representation of different age groups and demographic characteristics. While the use of semi-structured interviews as a qualitative method provided in-depth insights, the findings may have been influenced by personal biases and perspectives, potentially affecting their objectivity. The criterion sampling method, which selects participants based on specific characteristics, may have further constrained the generalizability of the findings to all individuals affected by the earthquake.

The exclusion of external factors such as psychological support systems, existing mental health conditions, or pre-earthquake physical activity levels may have overlooked other critical variables influencing exercise cravings. The data collection, conducted shortly after the earthquake, also limits the ability to assess long-term effects or changes over time. Although the inclusion of a vulnerable post-disaster population was ethically approved, participants’ emotional states might have affected their willingness to fully share their experiences, potentially impacting the reliability of the data.

Future research should include larger and more diverse samples to represent varying age groups and demographic characteristics. In addition to qualitative methods, employing quantitative methods would enhance the reliability and generalizability of the findings. Collecting data across different timeframes would allow comparisons of short- and long-term effects. Furthermore, adopting more structured and supportive approaches when working with vulnerable groups could improve data quality.

Studies should also incorporate not only sociodemographic variables but also physiological and morphological factors. For instance, variables such as participants’ physical health, musculoskeletal system characteristics, or biological responses to traumatic events should be included. Examining these factors would provide a more comprehensive understanding of the relationship between exercise cravings and post-traumatic behavioral responses. These recommendations will contribute to making future research in this area more robust and generalizable.

## 5 Conclusion

Similar to other substance and behavioral addictions, individuals with exercise addiction also exhibit craving behaviors. According to research findings, exercise craving manifests in two ways. The first type originates from exercise addiction, emerging when an individual with exercise addiction experiences a lack of exercise. The second type, with a more clinical dimension, arises from the inability to engage in training due to uncontrollable reasons. This form of craving is driven by concerns about the deterioration of the individual's current physical condition due to their inability to exercise. In the present study, an attempt was made to discern whether exercise craving exists based on participants’ narratives. This research is exploratory in nature. Therefore, for a more comprehensive measurement of the newly introduced concept of exercise craving in the field, the development of suitable measurement tools is recommended for future researchers.

## Statement of Ai

While preparing this work, the authors used Grammarly to improve readability and language. After using this tool, the authors reviewed and edited the content as needed and took full responsibility for the content of the publication.

## Data Availability

The data analyzed in this study is subject to the following licenses/restrictions: The data that support the findings of this study are available from the author, [II], upon reasonable request. Requests to access these datasets should be directed to Ismail Ilbak, isma_ilbak@hotmail.com.
